# Differential impact of the COVID-19 pandemic on excess mortality and life expectancy loss within the Hispanic population

**DOI:** 10.4054/DemRes.2023.48.12

**Published:** 2023-03-07

**Authors:** Elizabeth Arias, Betzaida Tejada-Vera

**Affiliations:** 1 National Center for Health Statistics, Hyattsville, Maryland, USA.; 2 National Center for Health Statistics, Hyattsville, Maryland, USA.

## Abstract

**BACKGROUND:**

The impact of the COVID-19 pandemic on the Hispanic population resulted in the almost complete elimination of the long-standing Hispanic mortality advantage relative to the non-Hispanic White population. However, it is unknown how COVID-19 mortality affected the diverse Hispanic subpopulations.

**OBJECTIVE:**

We estimate life expectancy at birth in 2019 and 2020 by select Hispanic country/region of origin and explore how changes in age-specific all-cause and COVID-19 mortality affected changes in life expectancy between 2019 and 2020 for each group.

**METHODS:**

We use final 2019 and 2020 mortality data from the National Center for Health Statistics and population estimates based on the 2019 and 2020 American Community Survey. We calculate life tables and apply decomposition techniques to explore the effects of changes in age- and cause-specific mortality on life expectancy.

**RESULTS:**

Patterns of age- and cause-specific excess deaths and their impact on declines in life expectancy due to the COVID-19 pandemic differed substantially by Hispanic subgroup. Life expectancy losses ranged from 0.6 to 6.7 years among males and from 0.6 to 3.6 years among females.

**CONCLUSIONS:**

Our findings highlight the heterogeneous impact of the COVID-19 pandemic within the Hispanic population.

**CONTRIBUTIONS:**

Our findings contribute new information that will help future researchers identify the causes of the disproportionately severe impact of the COVID-19 pandemic on the Hispanic population. Our study underscores the importance of population disaggregation in endeavors to identify the multiple pathways by which the pandemic affected the Hispanic population.

## 1. Introduction

The impact of the COVID-19 pandemic on the Hispanic/Latino (henceforth “Hispanic”) population has been disproportionate compared to other race/ethnicity groups (Macias Gil et al. 2020; Garcia et al. 2020; Saenz and Garcia 2020; Andrasfay and Goldman 2021; Arias et al. 2022). Until 2020 the Hispanic population experienced lower all-cause mortality than the non-Hispanic White population despite having lower levels of educational attainment, higher levels of poverty, and lower access to health insurance and quality care, a finding first observed more than 35 years ago (Markides and Coreil 1986; Markides and Eschbach 2011). In 2019 the Hispanic population had a life expectancy advantage relative to the non-Hispanic White population of more than three years (Arias and Xu 2022). In 2020 the advantage declined to half a year (Arias and Xu 2022).

Pre-pandemic, the Hispanic mortality advantage relative to the non-Hispanic White population was considerably greater among the foreign-born and varied by country/region of origin (Palloni and Arias 2004; Fenelon, Chinn, and Anderson 2017; Arias, Johnson, and Tejada-Vera 2020). Puerto Ricans experienced higher mortality than Cubans, Mexicans, and Central and South Americans (CSAs), and foreign-born CSAs, Dominicans, and Mexicans experienced the largest mortality advantages relative to the non-Hispanic White population (Fenelon, Chinn, and Anderson 2017; Arias, Johnson, and Tejada-Vera 2020). A recent study exploring the intersection of country of origin, nativity, and socioeconomic characteristics in COVID-19 mortality risks among Hispanics in California found the opposite of the pre-pandemic Hispanic mortality regime. Foreign-born Mexicans and Central Americans experienced considerably greater COVID-19 mortality than their US-born counterparts, particularly among working-age adults with low educational attainment and in unprotected essential jobs (Riley et al. 2021). These findings are of great value to our understanding of why the pandemic so disproportionately affected the Hispanic population. However, they pertain to only Hispanics of Mexican and Central American origin living in California. We do not know how other Hispanic subgroups in other parts of the country were impacted.

Life expectancy is a succinct measure of a population’s health status that is easily understood and comparable across populations and time periods. To explore the effects of the COVID-19 pandemic on mortality across diverse Hispanic country/region of origin populations in the United States, we estimate life expectancy at birth in 2019 and 2020 for Hispanic country of origin subgroups (Cuban, Mexican, Puerto Rican, CSA, and “other Hispanic”) by sex. Ideally, we would estimate life expectancy at birth by country of origin and nativity. However, due to data limitations, this is currently not possible. We explore how changes in age-specific all-cause and COVID-19 mortality affected changes in life expectancy between 2019 and 2020 for each subgroup in comparison to the non-Hispanic White population. We restrict the analysis to the first year of the COVID-19 pandemic due to the current unavailability of 2021 population data for Hispanic subgroups. This limitation does not bias our analysis because most of the negative effect of the pandemic on Hispanic life expectancy occurred in 2020 (Arias et al. 2022).

## 2. Methods

To estimate life expectancy at birth in 2019 and 2020 for Hispanic subgroups, we calculated complete period life tables using methodology developed by the National Center for Health Statistics (NCHS) to calculate annual life tables for the total Hispanic population. The methodology includes the use of age-specific classification ratios developed by NCHS to correct for Hispanic origin misclassification on death certificates and the application of statistical modeling to estimate mortality for the oldest ages (Arias, Heron, and Hakes 2016; Arias and Xu 2022). Overall, 3% of Hispanic decedents are misclassified on death certificates. Misclassification rates range from 0% among Cuban decedents to 21% among CSA decedents (Arias, Heron, and Hakes 2016). Classification ratios are available for the selected country/region of origin subgroups only by sex, which prevented the disaggregation of the CSA and “other Hispanic” subgroups or the disaggregation by country of origin and nativity. The “other Hispanic” group includes persons identifying as Dominican, Spanish, Latin American, Hispanic, or Latino/a.

We calculated ratios of the life table age-specific probabilities of dying in 2020 to those in 2019 and used Arriaga’s decomposition method to explore the effects of changes in age- and cause-specific mortality on the change in life expectancy between 2019 and 2020 (Arriaga 1984). The age-specific decomposition was done using five-year age groups, but the results are shown by broad age categories referring to children/adolescents (under 20), working-age adults (20–69), and older adults (70 and older).

We used 2019 and 2020 mortality data from NCHS and population estimates based on the full sample of the one-year 2019 American Community Survey (ACS) and the Public Use Microdata Sample (PUMS) of the one-year 2020 ACS (US Census Bureau 2019, 2020; National Center for Health Statistics 2020, 2021). Due to the impact of the COVID-19 pandemic, the US Census Bureau did not release the standard one-year ACS estimates for 2020. Race and Hispanic origin in NCHS mortality and ACS population data are based on the Office of Management and Budget’s 1997 “Revisions to the Standards for the Classification of Federal Data on Race and Ethnicity” (Office of Management and Budget 1997). To account for the sampling error introduced by ACS population estimates to the life expectancy estimates, we used the delta method to approximate the variances of the age-specific death rates used to calculate variances of the life table age-specific probabilities of death and life expectancies.

## 3. Results

### 3.1 Changes in life expectancy at birth

In 2019 the Hispanic population had a life expectancy at birth of 81.9 years (Table 1) (Arias and Xu 2022). Hispanic males and females had life expectancies of 79.1 and 84.4 years, respectively. Within the Hispanic population, life expectancy varied by as much as 6.1 years, with CSAs having the highest (84.4 years) and “other Hispanics” the lowest (77.9) life expectancy. Among males, the maximum difference was 7.7 years between CSAs and other Hispanics. Variation among females was lower, at 5.2 years, with Cuban females experiencing the highest (86.3 years) and “other Hispanic” females the lowest (81.1) life expectancy. Overall, the Hispanic population experienced a life expectancy advantage of 3.1 years relative to the non-Hispanic White population. The numbers varied from an advantage of 5.6 years for CSAs to a disadvantage of –0.9 years for “other Hispanics.” Among males, the variation went from an advantage of 6.0 years for CSAs to a disadvantage of –1.7 years for “other Hispanics.” The variation went from an advantage of 5.0 years for Cuban females to a disadvantage of –0.2 years for “other Hispanic” females.

In 2020 life expectancy declined by 4.0 years, to 77.9 years, for the Hispanic population (Table 1) (Arias and Xu 2022). Decreases in life expectancy varied significantly by subgroup. The CSA population experienced the greatest decline (–5.3 years) and “other Hispanics” the smallest (–0.6). Hispanic males lost 4.5 years of life expectancy between 2019 and 2020, with losses varying from 6.7 years for CSAs to 0.6 years for “other Hispanics.” Hispanic females lost 3.1 years, ranging across subgroups from 3.6 years for Mexicans to 0.6 years for “other Hispanics.” In 2020 the Hispanic mortality advantage was reduced to 0.5 years. It declined to 1.2 years for females and became a disadvantage of –0.2 years for males. Across the subgroups, only Cuban and CSA populations retained an advantage relative to the non-Hispanic White population in 2020 (3.1 and 1.7, respectively). Similarly, among males, only Cuban and CSA populations experienced advantages (2.9 and 0.8, respectively). Cuban, CSA, and Mexican females retained advantages of 3.1, 2.3, and 0.3, respectively. Among “other Hispanic” females, the disadvantage (–0.2) in 2019 became an advantage (0.4) in 2020.

### 3.2 Changes in age-specific mortality

Figures 1a–1b show ratios of the age-specific probabilities of dying in 2020 to those in 2019 for the non-Hispanic White, Hispanic, and Hispanic subgroups by sex for ages 20 to 100 and older. Due to very small numbers of deaths, ratios for ages under 20 vary widely and thus are not shown. The ratios show the large disparities in relative increases in mortality between the non-Hispanic White and the Hispanic populations. The age patterns of increases in mortality also differed between the two populations, especially among males. While the age pattern of mortality increases was relatively stable for non-Hispanic White males, for example, the pattern for Hispanic males demonstrated large increases and decreases with age, with the greatest increases in mortality in the age range 35 through 70.

There was considerable heterogeneity in the patterns and magnitude of the relative increases in age-specific mortality across Hispanic subgroups. CSA males experienced the greatest increases in mortality throughout ages 35 to 85, peaking at age 68, with mortality in 2020 two and a half times greater than in 2019. Mexican males followed with increases in mortality between ages 30 and 70 that were larger than those of the other subgroups. The patterns of age-specific increases in mortality for Puerto Rican and Cuban males were similar to each other and exhibited more variation than those of CSA and Mexican males. The age-specific pattern of mortality change for “other Hispanic” males was relatively constant, alternating with mortality increases and decreases of around 20% throughout most of the age span. The much larger increases in mortality observed for Hispanic males appear to be driven mainly by CSA male mortality followed by Mexican male mortality.

Among females, there was heterogeneity in the patterns and magnitude of age-specific changes in mortality, although not as large as for males. CSA females experienced the greatest increases in age-specific mortality in the middle to older ages, with a pronounced peak at age 67, where the probability of dying in 2020 was approximately two times greater than in 2019. Similar to their male counterparts, Mexican females followed, with greater increases in mortality compared to the other Hispanic subgroups. Patterns for Cuban and Puerto Rican females were similar to each other, with large increases in mortality in the younger adult ages. However, Cuban females experienced larger increases in mortality over age 80.

### 3.3 Effects of changes in age and cause-specific mortality

Table 2 presents the results of the decomposition of the effects of changes in age-specific all-cause mortality on the decline in life expectancy between 2019 and 2020 (Arriaga 1984). For all groups, the percent contribution of mortality increases in childhood/adolescence was minimal. The contribution of increases in working-age adult mortality was greater for males than females across all groups. Conversely, the effects of increases in mortality at ages 70 and older were greater among females across all groups. The age patterns of percent contributions to changes in life expectancy did not differ substantially between the overall Hispanic and non-Hispanic White populations, although the effects of increases in mortality for working-age adults were greater for the non-Hispanic White population than for the Hispanic population.

There is heterogeneity within the Hispanic population in the effects of age-specific increases in all-cause mortality. Among males, the percent contribution of mortality increases for working-age adults was greatest for Mexican (57.4%) and smallest for Cuban populations (39.5%). Conversely, the percent contribution of mortality increases for those aged 70 and older ranged from 41.3% for Mexican males to 58.4% for Cuban males. Among females, the percent contribution of mortality increases for working-age adults were smaller for “other Hispanic” and Cuban women (6.8% and 17.9%, respectively) compared to the other Hispanic subgroups. The percentages for Mexican, Puerto Rican, and CSA females were similar, at 38.5%, 38.4%, and 34.4%, respectively. The largest effects of age-specific increases in mortality occurred in the oldest ages for Cuban and “other Hispanic” females (81.4% and 87.1%, respectively). While still large, the contributions of increases in mortality in the oldest ages were lower for CSA, Mexican, and Puerto Rican females (65.5%, 60.8%, 59.1%, respectively).

**Figure 2:**
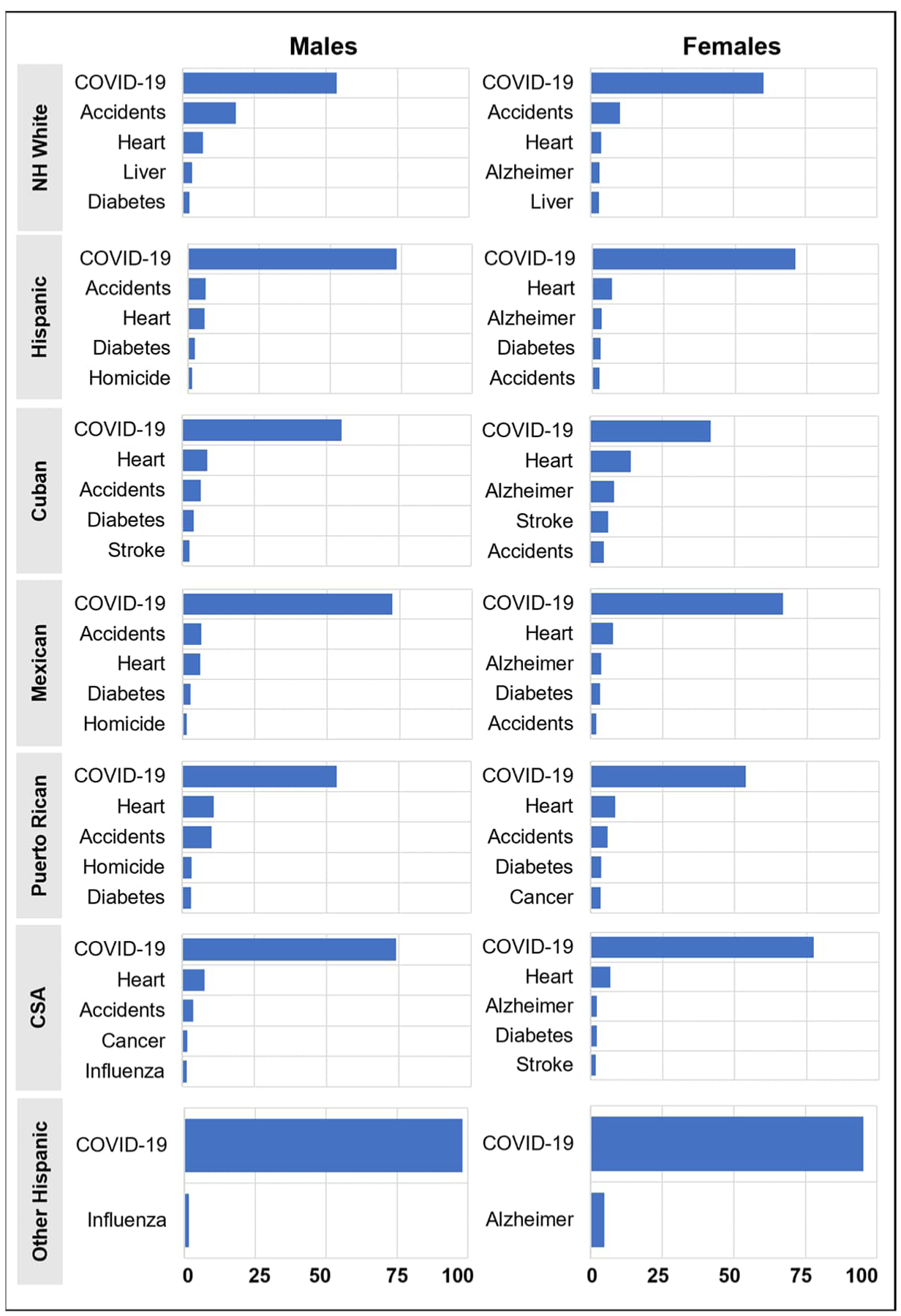
Percent contribution of increases in cause-specific mortality to declines in life expectancy at birth between 2019 and 2020 *Notes*: Race/ethnicity: non-Hispanic White (NH White), Central and South American (CSA). Causes of death: unintentional injuries (accidents), Alzheimer’s disease (Alzheimer), malignant neoplasms (cancer), coronavirus disease 2019 (COVID-19), diabetes mellitus (diabetes), diseases of the heart (heart), influenza and pneumonia (influenza), cerebrovascular diseases (stroke), chronic liver disease and cirrhosis (liver). *Source*: National Center for Health Statistics, National Vital Statistics System, Mortality.

Figure 2 shows the percent contribution of increases in the five causes of death that had the greatest impact on the decline in life expectancy between 2019 and 2020 (Arriaga 1984). Increases in mortality due to COVID-19 had the largest effect on declines in life expectancy in comparison to all other causes of death for both the Hispanic and non-Hispanic White populations. However, COVID-19 had a larger effect on the Hispanic population. Increases in mortality due to COVID-19 explained 73.3% and 71% of the decline in life expectancy among Hispanic males and females, respectively. In contrast, the effects for non-Hispanic White males and females were 53.8% and 60.5%, respectively.

There was substantial variation within the Hispanic population in the effects of increases in mortality due to COVID-19. The percent contributions of increases in mortality due to COVID-19 for males were smallest for Puerto Rican and Cuban men, at 53.6% and 55.3%, respectively, compared to 74.2% for CSA and 72.5% for Mexican males. The small decline in life expectancy (0.6 year) for “other Hispanic” males was almost entirely explained by COVID-19 mortality (98.2%). Cuban females experienced the smallest effect of increases in COVID-19 mortality on change in life expectancy (41.8%), while the effects for CSA and Mexican females were larger at 77.5% and 66.7%, respectively. As with their male counterparts, for “other Hispanic” females, COVID-19 explained most (95.3%) of the 0.6-year decline in life expectancy. There were some interesting differences across subgroups with respect to the effects of other causes of death. Notably, homicide was in the top five causes of death for Mexican and Puerto Rican males, and Alzheimer’s disease was in the top five for females in all subgroups except Puerto Ricans. We do not know at this time if and how increases in mortality from these other causes of death were related to the pandemic.

## 4. Conclusions

The goal of this study was to explore how excess mortality due to the COVID-19 pandemic differentially affected Hispanic country/region of origin subgroups. Results show that life expectancy loss varied greatly by Hispanic subgroup and sex. CSA and Mexican males experienced declines in life expectancy that were more than two times greater than those of other Hispanic males. Mexican and CSA females experienced the largest declines in life expectancy, but the variance across subgroups was much smaller than among males. “Other Hispanic” males and females experienced unusually small declines in life expectancy compared to the other Hispanic subgroups, but they had the lowest life expectancy and were the only subgroup that experienced a mortality disadvantage relative to the non-Hispanic White population before the pandemic.

The impact of increases in all-cause age-specific and COVID-19 mortality on the decline in life expectancy also differed greatly by subgroup. CSA and Mexican males experienced greater increases in mortality overall and in the working ages than did Cuban, Puerto Rican, and “other Hispanic” males. The decomposition revealed that increases in mortality in working ages had the largest effect on the decline in life expectancy for Mexican and CSA males. CSA and Mexican females experienced larger increases in mortality in the working ages, while Cuban and Puerto Rican females experienced greater increases in mortality at the oldest ages. The decomposition showed that increases in mortality in the oldest ages had the greatest effect on the decline in life expectancy for all Hispanic females, but with important differences across subgroups. Increases in mortality in the oldest ages explained most of the decline in life expectancy among Cuban females but explained substantially less for other females. Finally, the impact of increases in mortality due to COVID-19 on the decline in life expectancy also differed across Hispanic subgroups. The effect was considerably greater for CSA and Mexican males and females in comparison to Cuban and Puerto Rican males and females (aside from the “other Hispanic” population).

Our findings are partially consistent with those of the study that found that foreign-born Mexican and CSA males of working age in California experienced greater increases in mortality due to the COVID-19 pandemic than their US-born counterparts (Riley et al. 2021). Our results show that CSA and Mexican males experienced the largest declines in life expectancy at birth, mostly resulting from increases in mortality in the working ages. The life expectancy estimate for CSA males mostly reflects that of the foreign-born segment of this group, since 59.2% of CSA males were foreign-born. On the other hand, Mexican male life expectancy largely reflects that of the US-born segment (70.2%) of this population. Our findings lend support to the hypothesis that the excess burden of COVID-19 mortality for Mexican and CSA males may be explained by their overrepresentation in essential industries with higher risks of exposure to the virus (Do and Frank 2021; Billock, Steege, and Minino 2022; Goldman et al. 2021). However, for other Hispanic subgroups, particularly Cubans, other factors likely explain increases in mortality due to COVID-19. The decline in life expectancy among Cuban males and females was mostly a result of increases in mortality in the oldest ages. Perhaps the hypothesis that intergenerational transmission of the virus in multigenerational households explains at least partially the increase in mortality among Cubans (Do and Frank 2021). The Cuban population is majority foreign-born (56.2%) and is significantly older (median age 41 years) than the other subgroups, and multigenerational households have played a crucial role in this population’s socioeconomic adaptation in the United States (Arias 2001).

This study has important limitations. First, life expectancy estimates are based on mortality data that have been adjusted for Hispanic origin misclassification on death certificates and statistical modeling. As a result, the estimates are not free from error. Second, the “other Hispanic” and CSA subgroups combine diverse populations that likely have distinct mortality profiles. To test the validity of our subgroup life expectancy estimates, we estimated the life expectancy of the entire Hispanic population as the population weighted sum of the individual subgroup life expectancies. Ideally, our estimate should be very close to the official NCHS estimate. For 2019 our estimate was 0.2 years lower than the official estimate (81.7 versus 81.9), and for 2020 it was 0.5 years lower (77.4 versus 77.9). Despite these limitations, our study underscores the importance of population disaggregation in analyses of the multiple pathways by which the pandemic so disproportionately affected the Hispanic population.

## Figures and Tables

**Figure 1a:**
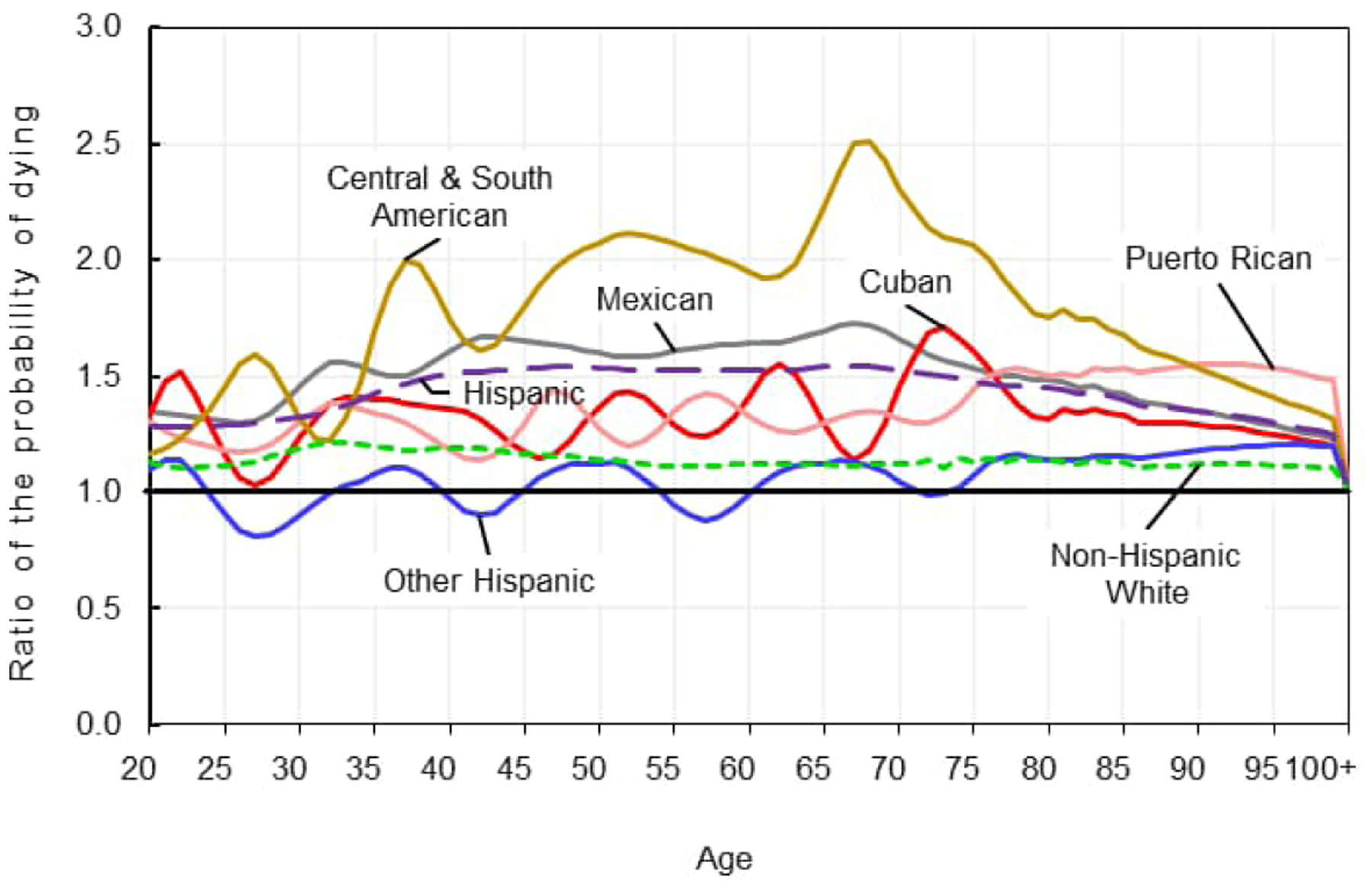
Age distribution of the relative increase in the probability of dying for males by Hispanic subgroup, 2019–2020 *Source*: National Center for Health Statistics, National Vital Statistics System, Mortality.

**Figure 1b:**
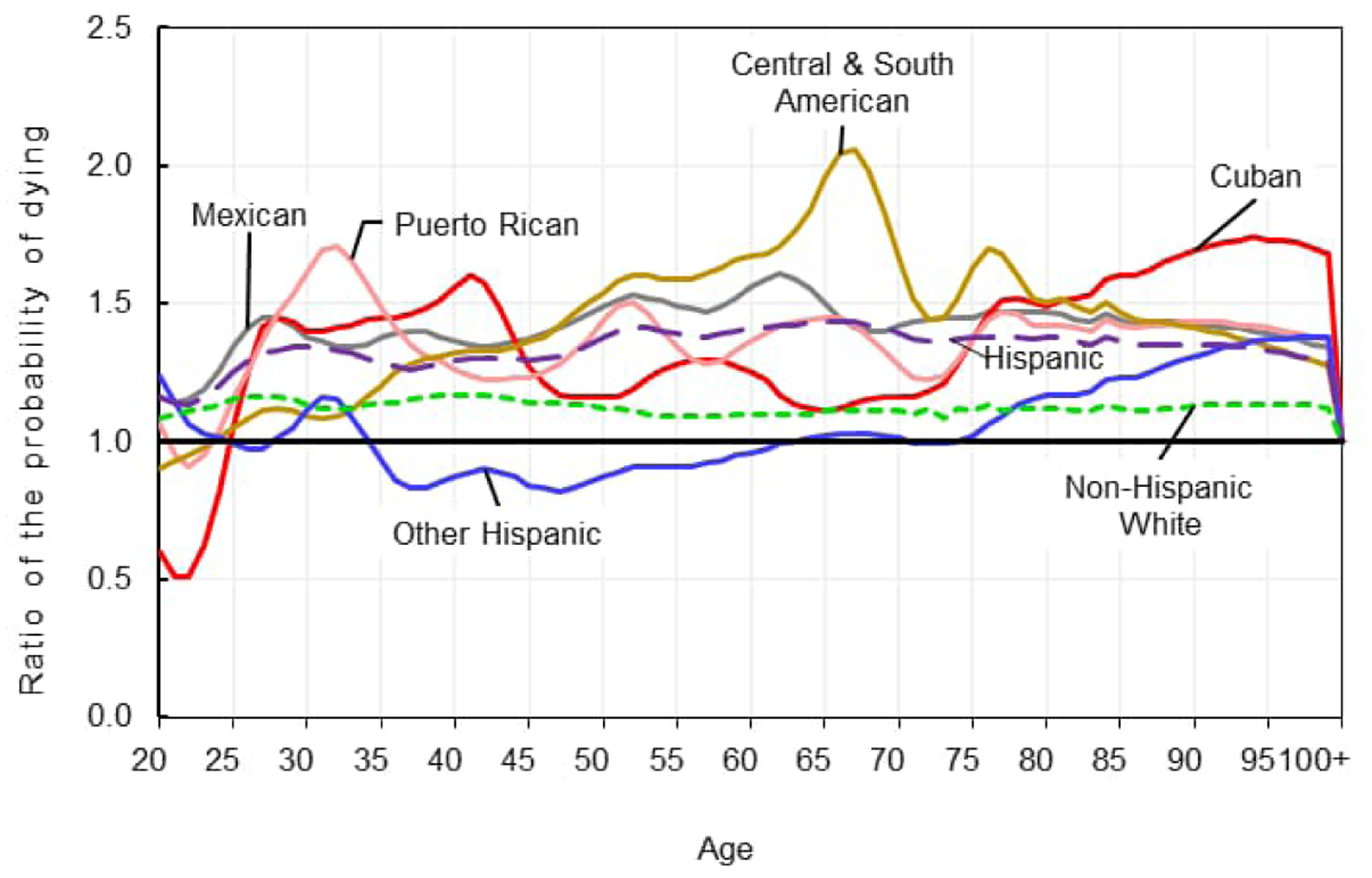
Age distribution of the relative increase in the probability of dying for females by Hispanic subgroup, 2019–2020 *Source*: National Center for Health Statistics, National Vital Statistics System, Mortality.

**Table 1:** Life expectancy at birth by Hispanic subgroup and sex, 2019 and 2020

	Life expectancy at birth (standard error)								
	Total			Males			Females		
	2019	2020	Difference	2019	2020	Difference	2019	2020	Difference
Hispanic	81.9	77.9	−4.0	79.1	74.6	−4.5	84.4	81.3	−3.1
Cuban	83.6 (.113)	80.5 (.099)	−3.1	80.7 (.169)	77.7 (.145)	−3.0	86.3 (.137)	83.2 (.116)	−3.1
Mexican	81.5 (.040)	76.9 (.038)	−4.6	78.8 (.060)	73.6 (.050)	−5.2	84.0 (.049)	80.4 (.040)	−3.6
Puerto Rican	80.2 (.089)	76.5 (.080)	−3.7	77.1 (.142)	73.3 (.107)	−3.8	83.0 (.120)	79.7 (.095)	−3.3
Central & South American	84.4 (.068)	79.1 (.061)	−5.3	82.3 (.108)	75.6 (.081)	−6.7	85.9 (.079)	82.4 (.077)	−3.5
Other Hispanic	77.9 (.099)	77.3 (.062)	−0.6	74.6 (.157)	74.0 (.091)	−0.6	81.1 (.119)	80.5 (.081)	−0.6
Non-Hispanic White	78.8	77.4	−1.4	76.3	74.8	−1.5	81.3	80.1	−1.2

*Notes*: “Other Hispanic” includes Dominicans, Spaniards, and persons identifying as Hispanic/Latino or Latin American.

*Source*: National Center for Health Statistics, National Vital Statistics System, Mortality.

**Table 2:** Percent contribution of increases in age-specific mortality to declines in life expectancy at birth between 2019 and 2020

	Males			Females		
	Under 20	20–69	70 and older	Under 20	20–69	70 and older
Hispanic	1.1	54.7	44.2	0.9	37.6	61.5
Cuban	2.0	39.5	58.4	0.7	17.9	81.4
Mexican	1.3	57.4	41.3	0.8	38.5	60.8
Puerto Rican	2.3	46.2	51.5	2.5	38.4	59.1
Central and South American	0.4	49.3	50.3	0.1	34.4	65.5
Other Hispanic	0.0	42.6	57.4	6.0	6.8	87.1
Non-Hispanic White	2.3	58.4	39.4	0.2	44.5	55.3

*Notes*: “Other Hispanic” includes Dominicans, Spaniards, and persons identifying as Hispanic/Latino or Latin American.

*Source*: National Center for Health Statistics, National Vital Statistics System, Mortality.
